# The Effect of Functional Groups on the Phase Behavior of Carbon Dioxide Binaries and Their Role in CCS

**DOI:** 10.3390/molecules26123733

**Published:** 2021-06-18

**Authors:** Sergiu Sima, Catinca Secuianu

**Affiliations:** 1 Department of Inorganic Chemistry, Physical Chemistry and Electrochemistry, Faculty of Applied Chemistry and Materials Science, University “Politehnica” of Bucharest, 1-7 Gh. Polizu Street, S1, 011061 Bucharest, Romania; sergiu.sima@upb.ro; 2 Department of Chemical Engineering, Imperial College London, South Kensington Campus, London SW7 2AZ, UK

**Keywords:** carbon dioxide, ethyl acetate, 1,4-dioxane, 1,2-dimethoxyethane, phase behavior, GEOS, PR EoSs, CCS

## Abstract

In recent years we have focused our efforts on investigating various binary mixtures containing carbon dioxide to find the best candidate for CO_2_ capture and, therefore, for applications in the field of CCS and CCUS technologies. Continuing this project, the present study investigates the phase behavior of three binary systems containing carbon dioxide and different oxygenated compounds. Two thermodynamic models are examined for their ability to predict the phase behavior of these systems. The selected models are the well-known Peng–Robinson (PR) equation of state and the General Equation of State (GEOS), which is a generalization for all cubic equations of state with two, three, and four parameters, coupled with classical van der Waals mixing rules (two-parameter conventional mixing rule, 2PCMR). The carbon dioxide + ethyl acetate, carbon dioxide + 1,4-dioxane, and carbon dioxide + 1,2-dimethoxyethane binary systems were analyzed based on GEOS and PR equation of state models. The modeling approach is entirely predictive. Previously, it was proved that this approach was successful for members of the same homologous series. Unique sets of binary interaction parameters for each equation of state, determined for the carbon dioxide + 2-butanol binary model system, based on *k*_12_–*l*_12_ method, were used to examine the three systems. It was shown that the models predict that CO_2_ solubility in the three substances increases globally in the order 1,4-dioxane, 1,2-dimethoxyethane, and ethyl acetate. CO_2_ solubility in 1,2-dimethoxyethane, 1.4-dioxane, and ethyl acetate reduces with increasing temperature for the same pressure, and increases with lowering temperature for the same pressure, indicating a physical dissolving process of CO_2_ in all three substances. However, CO_2_ solubility for the carbon dioxide + ether systems (1,4-dioxane, 1,2-dimethoxyethane) is better at low temperatures and pressures, and decreases with increasing pressures, leading to higher critical points for the mixtures. By contrast, the solubility of ethyl acetate in carbon dioxide is less dependent on temperatures and pressures, and the mixture has lower pressures critical points. In other words, the ethers offer better solubilization at low pressures; however, the ester has better overall miscibility in terms of lower critical pressures. Among the binary systems investigated, the 1,2-dimethoxyethane is the best solvent for CO_2_ absorption.

## 1. Introduction

Green chemistry, green engineering, and sustainable development have become strategic priority areas in both business and academia over the last decade. Chemical methods that produce goods with the least amount of waste and hazardous chemicals, coupled with cost improvements, have opened the door to innovative solutions. Special attention is paid to the re-examination of industrial fluids and solvents, and methods to produce the next generation of clean, healthy, reliable, and useful products and processes that are also good for human health and the environment [1,2,3,4].

Fluids such as carbon dioxide (CO_2_) and water are among the most naturally non-hazardous and stable substances we use in our everyday lives, and they will most likely be the working fluids of choice for the next wave of green goods and processes due to their environmental compatibility. Physical properties and phase behavior of complex CO_2_-containing mixtures are now correlated with a diverse range of applications [2,3,4].

Simultaneously, carbon (as CO_2_) emissions from a variety of processes, including fossil fuel-powered plants and electricity processing facilities, account for more than 80% of greenhouse gas emissions (GHGs) [5]. CO_2_ levels have steadily risen from 280 ppm to over 400 ppm since pre-industrial times [6]. Furthermore, even assuming steady emissions in the coming decades, the International Panel on Climate Change (IPCC) anticipated that CO_2_ concentrations in the atmosphere might reach at least 570 ppm by 2050, triggering a 2 °C rise in global mean temperature [7]. Reducing emissions by capturing CO_2_ at stationary point sources (e.g., power plants, refineries, oil and gas processing sites, iron and steel factories, cement plants, and other chemical plants) would only delay the rise in CO_2_ concentration in the atmosphere, and it is widely acknowledged that technologies that can extract CO_2_ at low concentrations are urgently needed. Carbon capture and storage (CCS) is a critical technology for the cost-effective mitigation of anthropogenic CO_2_ emissions, with the International Energy Agency (IEA) estimating that it may contribute around 20% to CO_2_ emission reductions by 2050 [8].

Alternatives to fossil fuels, such as nuclear, solar, wind, and biomass energies, are now being developed to prevent further global climate change, yet these energy sources will not be able to supply our modern society’s needs. Fossil fuels will continue to be the primary source of energy in the present and near future due to their availability, ease of transportation, competitiveness, and other factors; it will therefore be necessary to research and develop a highly effective CO_2_ separation and capture technology in order to meet CO_2_ reduction targets (CCS).

Chemical absorption, physical adsorption, membrane separation, cryogenic fractionation, and adsorption employing molecular sieves are the most widely utilized CO_2_ separation processes. Traditional technologies include limitations such as high energy consumption, chemical degradation, low/insufficient capacity, corrosion, foaminess, and so on [9,10,11,12]. Furthermore, according to recent studies [13,14] these approaches raise the energy requirements of power plants by 25–40%, with the separation process accounting for two-thirds of the entire CCS cost, even though some of them are energy efficient and environmentally friendly. CCS costs could be decreased in circumstances where industrial operations produce large amounts of CO_2_ gas streams or even pure CO_2_, but they remain a barrier to its application. Many studies in this area have concentrated almost entirely on the creation of improved sorbents with enhanced CO_2_ capacity and/or lower regeneration heat.

Recent findings [15,16] show that, while equilibrium CO_2_ capacity is a key determinant of process performance, phase equilibria, transport properties (e.g., viscosity, diffusion coefficients, etc.) and other thermophysical properties (e.g., heat capacity, density, etc.) all have a significant impact on the capital cost, and thus the price of carbon captured.

In addition, for optimal design and function, knowledge of the thermophysical properties and phase equilibria of systems of interest for CCS is a must.

In this context our group focused on investigating the phase behavior of carbon dioxide with various organic compounds as a way of carbon mitigation. The phase behavior (and thermophysical properties) of selected hydrocarbons (*n*-alkanes, branched alkanes, naphthenes) and functional group substances (e.g., alcohols, ethers, esters) were studied to determine their suitability as CO_2_ capture solvents/cosolvents [17,18,19,20,21,22,23]. Experiments are well known for being both costly and time consuming. Consequently, the alternative is the use of equations of state (EoS) models, that are the most widely used method for correlating and/or predicting phase equilibria and mixture properties, even though they have their known limitations [24]. Therefore, in this study we propose a predictive two-fold modelling approach, and we demonstrate that reasonably good qualitative results can be obtained based on a well-documented carbon dioxide containing system for other systems, if experimental data are not available for the latter. The model system we selected is the carbon dioxide + 2-butanol binary system, for which the complete experimental phase behavior is available [2,25]. The thermodynamic models chosen are the classic Peng–Robinson (PR) equation of state and the General Equation of State (GEOS), which is a generalization for all cubic equations of state with two, three, and four parameters, coupled with classical van der Waals mixing rules (two-parameter conventional mixing rule, 2PCMR). Both PR equation of state and GEOS were selected for their simplicity and availability.

In the current work, we present the results for three binary systems of carbon dioxide and some oxygenated organic compounds with four carbon atoms, namely ethyl acetate (EA), 1,4-dioxane (D), and 1,2-dimethoxyethane (DME). The two first compounds are isomers with the formula C_4_H_8_O_2_; ethyl acetate belonging to the esters class, while 1,4-dioxane to cyclic ethers; and the last one is a linear diether with the formula C_4_H_10_O_2_, as can be seen in Table 1.

The second modelling objective is to compare the predicted impact of functional group effect on the solvent ability to dissolve CO_2_, as part of our long-term project to identify the best candidate(s) as physical solvent for CO_2_ capture.

## 2. Modelling

Cubic equations of state have been extensively studied since van der Waals proposed his famous equation in 1873, and they are still the most popular method for the correlation and prediction of phase equilibria and mixture properties, with many practical applications [4,26]. They provide the best combination of precision, simplicity, reliability, and computation speed, and they continue to be effective and simple tools for calculating the phase behavior of many systems, including complex mixtures such as petroleum fluids, regardless of their known limitations [26,27,28].

The General Equation of State [29,30] with four parameters, and the Peng–Robinson equation of state [31] with two parameters, coupled with classical van der Waals mixing rules (two-parameter conventional mixing rules, 2PCMR) were chosen to investigate the phase behavior of the selected carbon dioxide containing mixtures.

The Peng–Robinson equation of state [31] is:

(1) $$P=\frac{RT}{V-b}-\frac{a\left(T\right)}{V\left(V+b\right)+b\left(V-b\right)}$$

where the two parameters, *a* and *b*, are:

(2) $$a=0.45724\frac{R^{2}{T_{c}}^{2}}{P_{c}}\alpha \left(T\right)$$

(3) $$b=0.077796\frac{RT_{c}}{P_{c}}$$

(4) $$\alpha \left(T_{R},\omega \right)={\left[1+m_{\mathrm{PR}}\left(1-{T_{R}}^{0.5}\right)\right]}^{2}$$

(5) $$m_{\mathrm{PR}}=0.37464-1.54226\omega -0.26992\omega^{2}$$

The cubic equation of state GEOS [29,30] has the form:

(6) $$P=\frac{RT}{V-b}-\frac{a\left(T\right)}{{\left(V-d\right)}^{2}+c}$$

The four *a*, *b*, *c*, and *d* parameters are given by the following relations for *a* pure component:

(7) $$\{\begin{array}{l} a\left(T\right)=a_{c}\beta \left(T_{r}\right)\;\;a_{c}=\Omega_{a}\frac{R^{2}{T_{c}}^{2}}{P_{c}}\;\;b=\Omega_{b}\frac{RT_{c}}{P_{c}}\\ c=\Omega_{c}\frac{R^{2}{T_{c}}^{2}}{{P_{c}}^{2}}\;\;d=\Omega_{d}\frac{RT_{c}}{P_{c}} \end{array}$$

The temperature function used in cubic GEOS is:

(8) $$\beta \left(T_{r}\right)={T_{r}}^{-m}$$

where *T_r_* is the reduced temperature, $T_{r}=T/T_{c}$. The expressions of the *Ω_a_*, *Ω_b_*, *Ω_c_*, and *Ω_d_* parameters are:

(9) $$\Omega_{a}={\left(1-B\right)}^{3};\;\Omega_{b}=Z_{c}-B;\;\Omega_{c}={\left(1-B\right)}^{2}\left(B-0.25\right);\;\Omega_{d}=Z_{c}-\frac{\left(1-B\right)}{2}$$

with

(10) $$B=\frac{1+m}{\alpha_{c}+m},\;\mathrm{where}\;\alpha_{c}.\;\mathrm{is}\;\mathrm{Riedel}’s\;\mathrm{criterion}.$$

The *a*, *b*, *c*, and *d* coefficients in GEOS are in fact functions of the critical data (*T_c_*, *P_c_*, and *V_c_*), *m*, and *α_c_* parameters. We should mention that GEOS is a general form for all cubic equations of state with two, three, and four parameters, as previously demonstrated [32]. Thus, the parameters of the Peng–Robinson equation of state can be obtained from the Equations (7)–(9) by setting the following restrictions: $\Omega_{c}=-{\left(\frac{\Omega_{b}}{2}\right)}^{2}$ and $\Omega_{d}=-\frac{\Omega_{b}}{2}$ respectively. It follows that:

(11) $$B=0.25-\frac{1}{8}{\left(\frac{1-3B}{1-B}\right)}^{2}\;\mathrm{and}\;Z_{c}\left(PR\right)=\frac{1+B}{4}$$

resulting B(PR)=0.2296 and $Z_{c}\left(PR\right)=0.3074$. The original temperature function *β*(*T_r_*) was used for PR EoS [31].

The first Equation (11) for *B* can be solved iteratively, starting with an initial approximation of *B* in the right-hand term. The corresponding values for *Ω_a_*, *Ω_b_*, *Ω_c_*, and *Ω_d_* are given in Equation (9), and are shown in Table 2 for GEOS, as they are compound-dependent for it, while for PR they are universal (*Ω_a_* = 0.4572; *Ω_b_* = 0.0778; *Ω_c_* = −0.0121; *Ω_d_* = −0.0778). It must also be mentioned that the values of *Z_c_* in GEOS are the experimental values [33].

The coefficients *a*, *b*, *c*, and *d* were obtained for mixtures using the classical van der Waals two-parameter conventional mixing rules (2PCMR for *a*, *b*) extended correspondingly for *c* and *d*:

(12) $$a=\sum_{i}\sum_{j}x_{i}x_{j}a_{ij};\;b=\sum_{i}\sum_{j}x_{i}x_{j}b_{ij};\;c=\sum_{i}\sum_{j}x_{i}x_{j}c_{ij};\;d=\sum_{i}x_{i}d_{i}$$

(13) $$a_{ij}={\left(a_{i}a_{j}\right)}^{1/2}\left(1-k_{ij}\right);\;b_{ij}=\frac{b_{i}+b_{j}}{2}\left(1-l_{ij}\right)$$

$c_{ij}=\pm {\left(\left|c_{i}\right|\left|c_{j}\right|\right)}^{1/2}$ (with “+” for *c_i_*, *c_j_* > 0 and “−” for *c_i_* or *c_j_* < 0).

Generally, negative values are common for the *c* parameter of pure components. The geometric mean in Equation (13) for *c_ij_* was explained in previous papers [34,35]. For PR EoS *a* and *b* are given by Equations (12) and (13) and *c* and *d* are calculated by the restrictions *c* = −2*b*^2^ and *d* = −*b* [32].

The GEOS parameters *m* and *α_c_* of each component were estimated by constraining the EoS to reproduce the experimental vapor pressure and liquid volume on the saturation curve between the triple point and the critical point. The values of critical data and GEOS parameters of the pure components are given in Table 3. The critical data and acentric factors of pure components are taken from the DIPPR database [33].

The calculations were made using our in-house software package PHEQ (Phase Equilibria Database and Applications) [36], and GPEC (Global Phase Equilibrium Calculations) [37,38,39]. The CRITHK module in our software uses the method designed by Heidemann and Khalil [40] for calculating the critical curve, where the numerical derivatives given by Stockfleth and Dohrn [41] are implemented.

## 3. Results and Discussion

Models capable of predicting equilibria properties without the use of experimental data, which yield accurate results in both the sub-critical and critical regions, are needed in modern process design.

Although correlating experimental data on a limited range of pressures, temperatures, and compositions is the preferred method by many research groups, efforts were dedicated to analyzing the phase behavior of systems using a global approach [17,18,19,20,21,22,23,34,35,37,38,39,42]. In a previous study [34] we predicted the phase behavior of the carbon dioxide + 2-butanol binary system, as extensive experimental data were available, using the *k*_12_–*l*_12_ method [42]. Thus, in a broad range of temperatures, we calculated a unique set of interaction parameters that accurately represented the experimental critical pressure maximum (CPM) and the experimental temperature of the upper critical endpoint (UCEP) of the system with different equations of state [34]. The carbon dioxide + 2-butanol system is a type II phase diagram [43,44] since it exhibits liquid–liquid immiscibility [45]. As a function of two independent variables, phase diagrams show the domains occupied by the various phases of a system, the boundaries that distinguish these areas, and the special points of the system [43,44,46]. As in type I phase behavior, type II phase behavior is characterized by a continuous liquid–vapor (LV) critical curve extending between the critical points of the pure components, but also by a liquid–liquid (LL) critical curve intersecting the three-phase liquid–liquid–vapor equilibrium line (LLV) in an upper critical endpoint (UCEP) [43,44]. Furthermore, in a previous paper [47] using the unique sets (*k*_12_, *l*_12_) of binary interaction parameters (BIPs) calculated for carbon dioxide + 2-butanol system with the Soave–Redlich–Kwong (SRK) [48] and Peng–Robinson (PR) [31] equations of state, we successfully predicted the phase behavior of the carbon dioxide + 2-propanol system. The organic compound, 2-propanol (C_3_H_8_O) belongs to the same homologous class of secondary alcohols.

In this study, we are using the sets obtained with GEOS (*k*_12_ = 0.050; *l*_12_ = −0.040) and PR EoS (*k*_12_ = 0.025; *l*_12_ = −0.108) for the carbon dioxide + 2-butanol system [34] to predict the phase behavior of carbon dioxide + ethyl acetate (EA), + 1,4-dioxane (D), and + 1,2-dimethoxyethane (DME). Although 2-butanol (C_4_H_10_O) and the organic compounds selected as the second component in the binary systems for this analysis are not isomers, they have in common four carbon atoms, 8 to 10 hydrogen atoms, and one or two oxygen atoms.

The critical pressures of pure ethyl acetate and 1,2-dimethoxyethane are very close (Table 3), followed by 2-butanol (41.89 bar [33]) and 1,4-dioxane; while the critical temperatures are increasing in the order ethyl acetate, 2-butanol (535.90 K [33]), 1,2-dimethoxyethane, and 1,4-dioxane.

**Figure 1:**
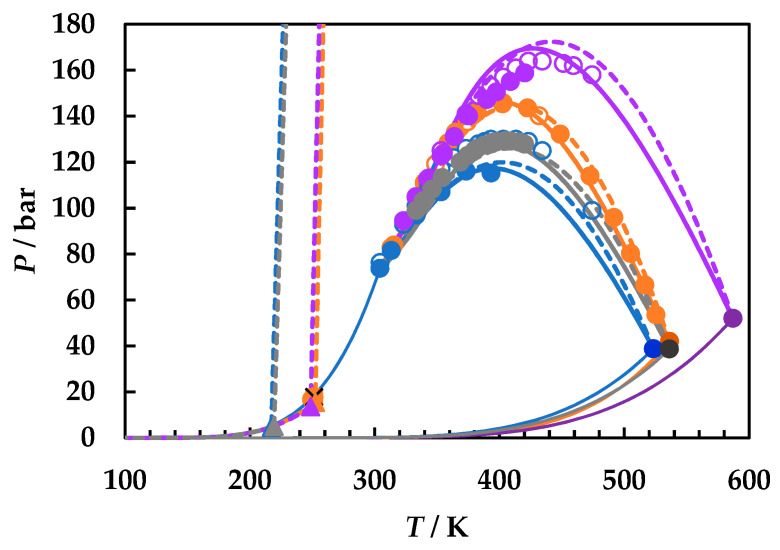
*P*–*T* fluid phase diagrams for carbon dioxide (1) + ethyl acetate (2), + 1,2-dimethoxyethane (2), + 2-butanol (2), and + 1,4-dioxane (2) binary systems. Symbols, literature data [22,45,50,51,52,53]; full lines, predictions by GEOS; dotted lines, predictions by Peng–Robinson (PR) equation of state (EoS). CO_2_ + ethyl acetate (**blue**); CO_2_ + 1,2-dimethoxyethane (**gray**); CO_2_ + 2-butanol (**orange**); CO_2_ + 1,4-dioxane (**purple**).

**Figure 2:**
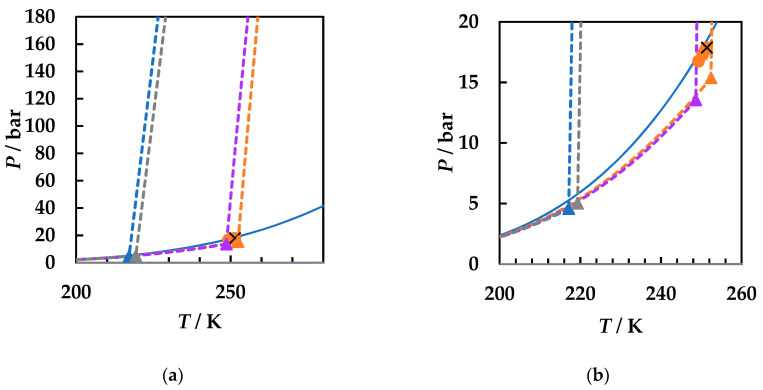
*P*–*T* fluid phase diagrams for carbon dioxide (1) + ethyl acetate (2), + 1,2-dimethoxyethane (2), + 2-butanol (2), and + 1,4-dioxane (2) binary systems calculated with PR EoS. (**a**) Detail of the predicted liquid–liquid critical lines, upper critical endpoints, and three-phase equilibrium lines. (**b**) Enlargement of the three-phase region. PR EoS predictions: dashed lines and triangles. Experimental data: cross (UCEP), circles, and square [45]. CO_2_ + ethyl acetate (**blue**); CO_2_ + 1,2-dimethoxyethane (**gray**); CO_2_ + 2-butanol (**orange**); CO_2_ + 1,4-dioxane (**purple**).

**Figure 3:**
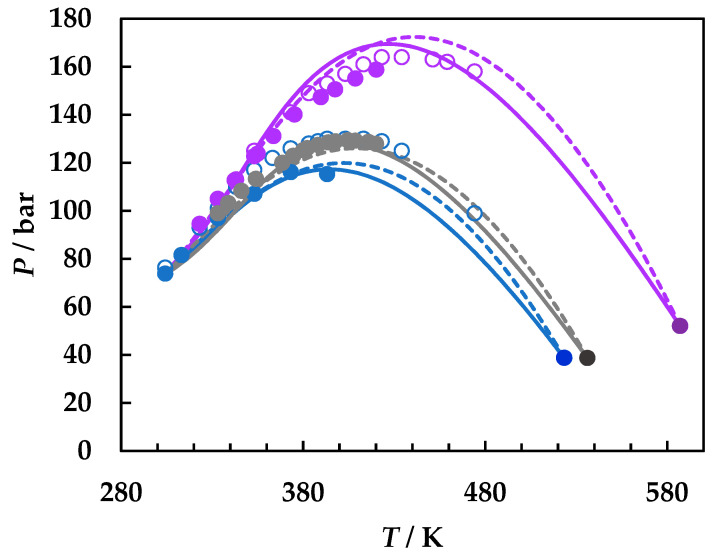
*P*–*T* fluid phase diagrams for carbon dioxide (1) + ethyl acetate (2), + 1,2-dimethoxyethane (2), and + 1,4-dioxane (2) binary systems, detailing the LV critical curves. Symbols, literature data [22,49,50,52]; full lines, predictions by GEOS; dotted lines, predictions by PR EoS. CO_2_ + ethyl acetate (**blue**); CO_2_ + 1,2-dimethoxyethane (**gray**); CO_2_ + 1,4-dioxane (**purple**).

A careful literature search was performed [2,49] and all available literature data for the three binary systems were collected. Figure 1 presents the calculated phase diagrams for the chosen binaries, both with GEOS and PR EoS, as well as for the reference system carbon dioxide + 2-butanol. PR EoS predicts type II phase behavior for all systems. The three-phase liquid–liquid–vapor region and liquid–liquid critical lines are enlarged in Figure 2, while the liquid–vapor critical lines are detailed in Figure 3.

Figure 2a shows the liquid–liquid critical lines that intersect the three-phase liquid–liquid–vapor equilibrium curves in upper critical endpoints. The predicted UCEP temperatures are ranging increasingly in the order ethyl acetate, 1,2-dimethoxyethane, 1,4-dioxane, and 2-butanol. All predicted LL critical lines have positive slopes, translating in LL phase splitting in isothermal pressure–composition diagrams [43,44] and LL critical points at temperatures lower than the UCEP’s temperature. In Figure 2b the distribution of the predicted UCEPs is shown in detail, as well as the comparison of the experimental UCEP and three-phase equilibrium line [45] for carbon dioxide + 2-butanol with the predictions by PR EoS. The agreement between experimental data and predictions is remarkably good. It must be pointed out that although there is no experimental evidence that the carbon dioxide + ethyl acetate, + 1,4-dioxane, and + 1,2-dimethoxyethane are type II phase diagrams, the fact that the model predicts the UCEPs at low temperatures is an advantage for applications. Another observation is that GEOS correctly predicts type II phase behavior for the carbon dioxide + 2-butanol system and type I phase behavior for the systems under investigation. However, from the LV critical lines in Figure 3, it is clearly seen that the critical locus of the binary mixtures increases as the critical temperature of the more volatile component is increasing (EA < DME < D). Although ethyl acetate and 1,4-dioxane are isomers, the behavior of the carbon dioxide mixture seems to be dictated by the critical properties of pure components, which in this case are more similar between ethyl acetate and 1,2-dimethoxyethane, as well as the linear structures. It must also be noted that the CPM shifts to higher temperatures in the same order: carbon dioxide + ethyl acetate, + 1,2-dimethoxyethane, + 1,4-dioxane, from ~400 K to ~440 K, this asymmetrical shape of the LV critical curves being the most common one among binary systems [44].

**Figure 4:**
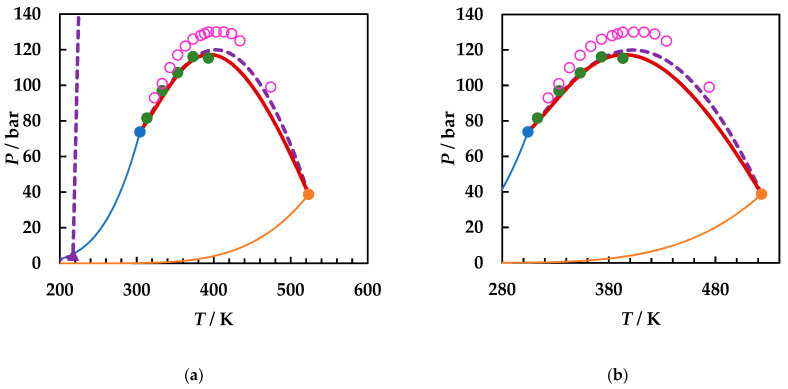
*P*–*T* fluid phase diagrams for carbon dioxide (1) + ethyl acetate (2) binary system. ●, [51]; ○, [50]; ●, ●, critical points of CO_2_, EA [33]; ─, ─, vapor pressures of pure components; ▬, GEOS predictions; ▲ (UCEP), - - - , PR predictions.

**Figure 5:**
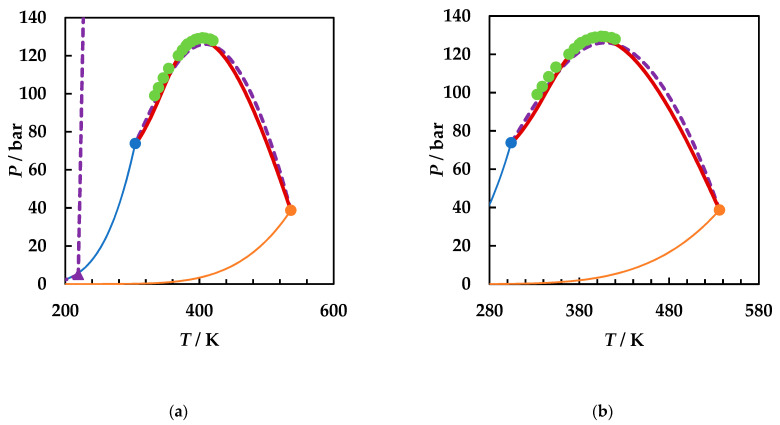
*P*–*T* fluid phase diagrams for carbon dioxide (1) + 1,2-dimethoxyethane (**2**) binary system. ●, [22]; ●, ●, critical points of CO_2_, EA [33]; ─, ─, vapor pressures of pure components; ▬, GEOS predictions; ▲, - - -, PR EoS predictions.

**Figure 6:**
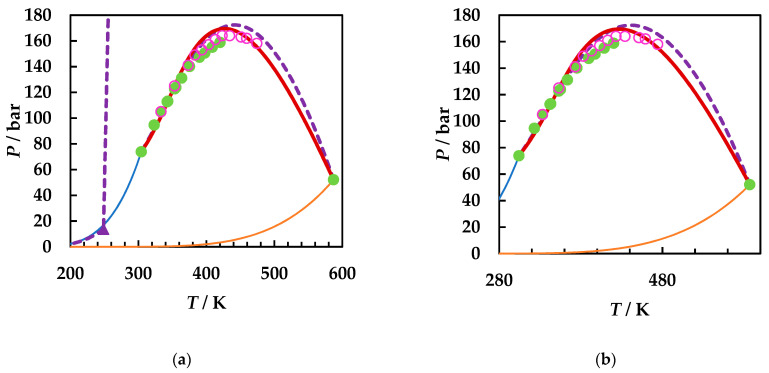
*P*–*T* fluid phase diagrams for carbon dioxide (1) + 1,4-dioxane (2) binary system. ●, [52]; ○, [49]; ●, ●, critical points of CO_2_, EA [33]; ─, ─, vapor pressures of pure components; ▬, GEOS predictions; ▲, - - -, PR EoS predictions.

Figure 4, Figure 5 and Figure 6 compare the predictions by GEOS and PR EoS for each binary system analyzed. Thus, Figure 4 shows the comparison of the available experimental critical data and the predictions by both GEOS and PR equation of state for the carbon dioxide + ethyl acetate binary system.

Figure 4a shows the entire phase diagram, including the predicted UCEP by PR EoS, while Figure 4b presents the enlargement of LV critical region. A clear dispersion of the experimental critical data is observed, the difference in CPM being more than 15 bar, while the divergence in corresponding temperatures is ~50 K. The experimental data from [51] are better predicted by both GEOS and PR EoS. However, the predicted CPM by GEOS is closer to the experimental one, including the corresponding temperature, while PR EoS predicts the CPM at a higher pressure (~2 bar larger), and more importantly, at a higher temperature (~20 K). On the other hand, both equations underpredict the critical curve at temperatures lower than ~350 K compared with the experimental data.

Figure 5 and Figure 6 are organized in the same way as Figure 4, displaying on the left side (a) the entire phase diagram and the enlargement of the liquid–vapor critical region, and on the right side (b) for the carbon dioxide + 1,2-dimethoxyethane and carbon dioxide + 1,4-dioxane, respectively. The predictions by both PR EoS and GEOS are quite accurate for the carbon dioxide + 1,2-DME binary system, for which only one set of experimental critical data is available [22], with a slight superiority by GEOS in the critical pressure maximum area (Figure 5), but with a pronounced underestimation in the LV critical region towards the critical point of carbon dioxide. The available experimental critical data [50,53] for the carbon dioxide + 1,4-dioxane binary system are in good concordance, with a slight discrepancy in the maximum critical pressure area. GEOS performs better than PR EoS for the carbon dioxide + 1,4-dioxane binary system, though the same trend as for the other two systems can be noticed—an underestimation of the critical points in the first part of the LV critical curve starting from the critical point of CO_2_, followed by a faster reach of the critical pressure maximum than the experimental data. PR EoS predicts the CPM at higher pressure and temperature than the experimental ones.

**Figure 7:**
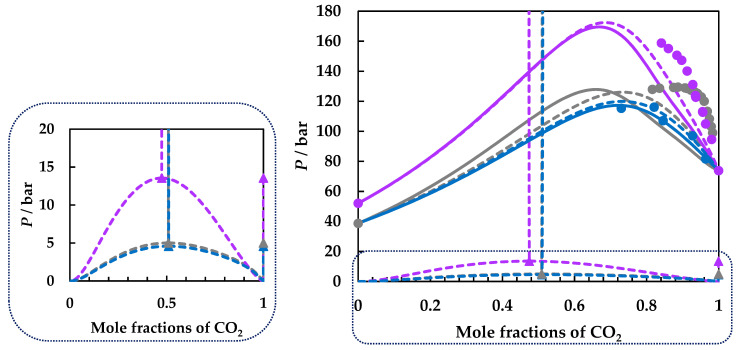
*P*–*X* fluid phase diagram of the carbon dioxide + ethyl acetate (**blue**), carbon dioxide + 1,4-dioxane (**purple**), and carbon dioxide + 1.2-dimethoxyethane (**gray**) binary systems. Symbols, literature data [22,51,53]; full lines, predictions by GEOS; dashed lines, predictions by PR EoS.

Although the pressure–temperature projections of the phase diagrams are reasonably well predicted considering that the BIPs have been determined for carbon dioxide + 2-butanol binary system, the pressure–composition projection predictions by both GEOS and PR EoS show qualitative agreement for CO_2_ + 1,4-dioxane and CO_2_ + 1,2-dimethoxyethane binary systems and display a better concordance for the CO_2_ + ethyl acetate system, but the experimental data are quite scattered for the latter. In Figure 7 are plotted the experimental data for the three binary systems studied, together with the predictions by GEOS and PR EoS. As PR EoS predicts type II phase behavior, the LL critical lines, the UCEPs, and the three-phase equilibrium lines are also shown. The three-phases region is additionally detailed on the left side of the figure. This behavior will be negatively reflected in isothermal phase diagrams as underestimation or overestimation of the carbon dioxide compositions, depending on the temperature.

**Figure 8:**
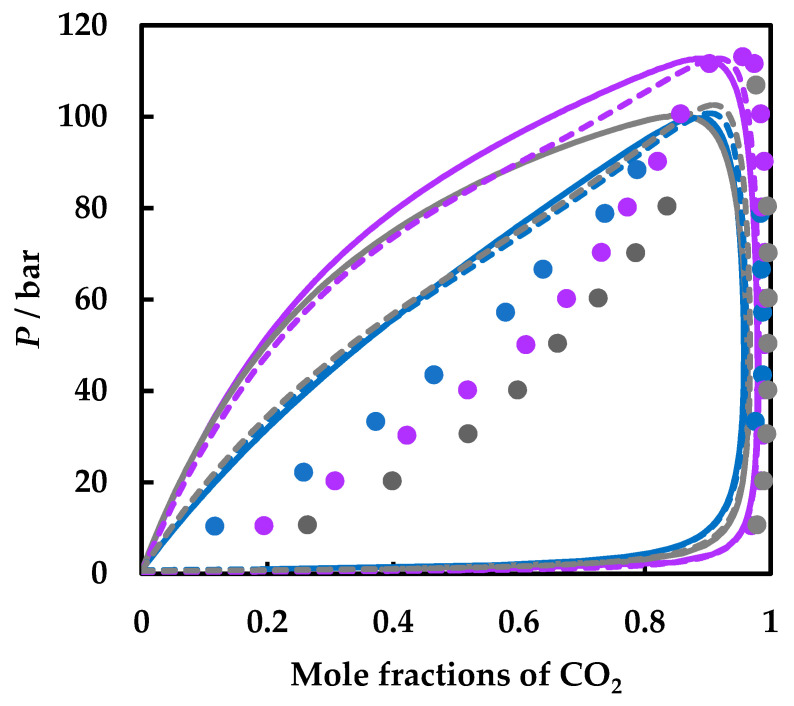
Comparison of literature data for CO_2_ + ethyl acetate (blue) [54], + 1,4-dioxane (purple) [53], and + 1,2-dimethoxyethane (**gray**) [22] binary systems at *T* = 343.15 K and predictions by GEOS (**full lines**) and PR EoS (**dashed lines**).

Thus, we compare the predictions by both GEOS and PR EoS with experimental isothermal pressure–composition data at 343.15 K for all three systems considered in Figure 8. This temperature is the highest common one for all systems for which experimental data are available. It must be mentioned that the fewest data are available for the carbon dioxide + 1,2-dimethoxyethane system [22]. Both models predict a much higher content of the organic compounds in the liquid phase, which increases with pressure, consistent with the behavior observed in the pressure–temperature and pressure–composition projections. The vapor phase is better predicted, though the models underestimate the carbon dioxide compositions.

**Figure 9:**
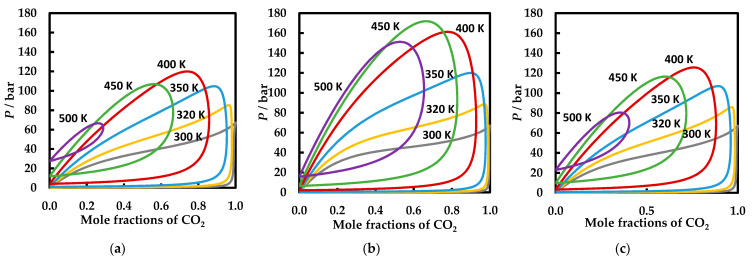
VLE behavior at six temperatures (300, 320, 350, 400, 450, and 500 K) for the carbon dioxide + ethyl acetate (**a**), carbon dioxide + 1,4-dioxane (**b**), and carbon dioxide + 1,2-dimethoxyethane (**c**) binary systems. The lines correspond to PR EoS predictions with the BIPs *k*_12_ = 0.025 and *l*_12_ = −0.108.

Note that at this temperature, the experimental data suggest that CO_2_ is most soluble in 1,2-dimethoxyethane, followed by 1,2-dioxane, and ethyl acetate, while the predictions indicate the order ethyl acetate, 1,2-dimethoxyethane, and 1,4-dioxane. However, the predictions become more accurate at higher temperature and pressures. For this purpose, we present PR EoS predictions at the same temperatures (300, 320, 350, 400, 450, and 500 K) for the three systems studied in Figure 9. The composition windows for phase separation at a specified temperature and pressure grow apparently in the order carbon dioxide + ethyl acetate (Figure 9a), carbon dioxide + 1,2-dimethoxyethane (Figure 9c), and carbon dioxide + 1,4-dioxane (Figure 9b). In other words, and more related to possible applications in CCS/CCUS, it means that the models predict that carbon dioxide is most soluble in ethyl acetate, followed by 1,2-dimethoxyethane, and 1,4-dioxane.

**Figure 10:**
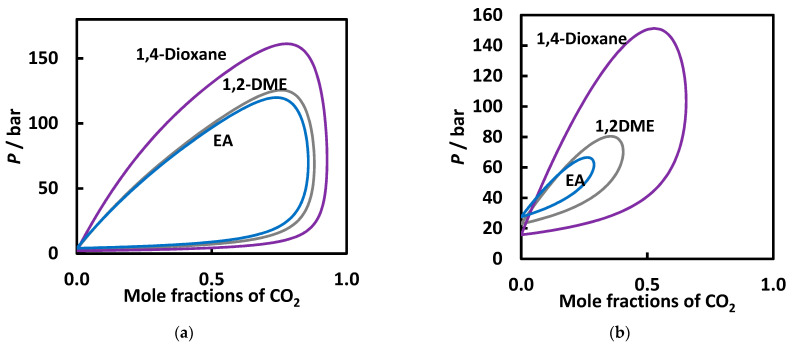
VLE behavior at 400 K (**a**) and 500 K (**b**) for the carbon dioxide + ethyl acetate, carbon dioxide + 1,4-dioxane, and carbon dioxide + 1,2-dimethoxyethane binary systems. The lines correspond to PR EoS predictions with the BIPs *k*_12_ = 0.025 and *l*_12_ = −0.108.

When comparing the prediction results at the same temperature for the three mixtures, it can be observed that at high temperatures and pressures, the CO_2_ solubility increases in the same order as the experiments suggest, i.e., 1,4-dioxane < ethyl acetate < 1,4-dimethoxyethane (Figure 10a). At higher temperatures and pressures, the differences become more conspicuous (Figure 10b). Considering solvent-solvent and solvent-CO_2_ interactions, it was demonstrated [55] that ether bonds in solvents can promote the CO_2_ absorption and perform better than esters. Moreover, the present study indicates that the oxygenated cyclic compound has the poorest solubility in CO_2_, confirming the same result when comparing cyclic alkanes and normal alkanes [17].

Further investigations are necessary to determine which class of organic substances is the best solvent for carbon dioxide capture.

## 4. Conclusions

The phase behavior of carbon dioxide + ethyl acetate, + 1,4-dioxane, and + 1,2-dimethoxyethane binary systems was reasonably well predicted using unique sets of binary interaction parameters tailored for the carbon dioxide + 2-butanol system using the *k*_12_-*l*_12_ method with GEOS (*k*_12_ = 0.050; *l*_12_ = −0.040) and PR EoS (*k*_12_ = 0.025; *l*_12_ = −0.108) models. Although the test is severe, both models show a very good agreement for the pressure–temperature phase diagrams for all three systems investigated. The carbon dioxide + 1,4-dioxane binary system displays the larger ranges of immiscibility, followed by the carbon dioxide + ethyl acetate, and carbon dioxide + 1,2-dimethoxyethane. The models predict that the most suitable candidate for CO_2_ capture among the three binary mixtures considered is carbon dioxide + 1,2-dimethoxyethane. These results show that, based on similarity of compounds, qualitative information can be obtained without having experimental data for the systems under investigation. It was shown that linear ethers are the most favorable solvents, followed by esters, and cyclic esters. Further studies are needed to determine if the members of the same organic family behave similarly, and which class of organic substances is the most suitable as a physical solvent.

## Figures and Tables

**Table 1:** Compound structures and molecular mass.

Compounds	Formula	Chemical Structure	Molecular Weight	CAS Number
Carbon dioxide	CO_2_	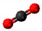	44.0095	124-38-9
Ethyl acetate	C_4_H_8_O_2_	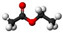	88.1051	141-78-6
1,4-Dioxane	C_4_H_8_O_2_	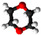	88.1051	123-91-1
1,2-dimethoxyethane	C_4_H_10_O_2_	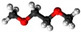	90.1210	110-71-4

**Table 2:** The critical compressibility factor (*Z_c_*), and General Equation of State (GEOS) parameters (*B*, *Ω_a_*, *Ω_b_*, *Ω_c_*, *Ω_d_*).

Substance	CO_2_	EA	D	DME
*B*	0.1767	0.1720	0.1612	0.1475
*Z_c_*	0.2740	0.2550	0.2540	0.2305
*Ω_a_*	0.5582	0.5677	0.5903	0.6196
*Ω_b_*	0.0973	0.0830	0.0928	0.0875
*Ω_c_*	−0.0497	−0.0535	−0.0625	−0.0745
*Ω_d_*	−0.1377	−0.1590	−0.1654	−0.1913

**Table 3:** Critical data (*T_c_*, *P_c_*, *V_c_*), acentric factor (*ω*) [33], and GEOS parameters (*α_c_*, *m*) for pure compounds.

Compounds	*T_c_*/K	*P*_c/_bar	*V_c_*/cm^3^·mol^−1^	*ω*	*α*_c_	*m*
CO_2_	304.21	73.83	94.0	0.2236	7.0801	0.3045
EA	523.30	38.80	286.0	0.3664	7.9337	0.4403
D	587.00	52.08	238.0	0.2793	7.8346	0.3130
DME	536.15	38.71	270.6	0.3475	8.6973	0.3315
